# A Thixotropic Polyglycerol Sebacate-Based Supramolecular Hydrogel as an Injectable Drug Delivery Matrix

**DOI:** 10.3390/polym8040130

**Published:** 2016-04-07

**Authors:** Hongye Ye, Cally Owh, Shan Jiang, Cavin Zhen Quan Ng, Daniel Wirawan, Xian Jun Loh

**Affiliations:** 1 Institute of Materials Research and Engineering (IMRE), A*STAR (Agency for Science, Technology and Research), 2 Fusionopolis Way, Innovis, #08-03, Singapore 138634, Singapore; yehy@imre.a-star.edu.sg (H.Y.); cally-owh@imre.a-star.edu.sg (C.O.); summerjiangshan@hotmail.com (S.J.); 2 College of Chemistry, Jilin University, Changchun 130012, China; 3 Department of Materials Science and Engineering, National University of Singapore, 9 Engineering Drive 1, Singapore 117575, Singapore; a0108312@u.nus.edu; 4 Department of Biomedical Engineering, National University of Singapore, 4 Engineering Drive 3, Singapore 117583, Singapore; a0099428@u.nus.edu

**Keywords:** polyglycerol sebacate, shear-thinning, self-healing, injectable hydrogel, supramolecular

## Abstract

We have developed a “self-healing” polyglycerol sebacate—polyethylene glycol methyl ether methacrylate (PGS-PEGMEMA)/α-Cyclodextrin (αCD) hydrogel which could be sheared into a liquid during injection and has the potential to quickly “heal” itself back into gel post-injection. This hydrogel was shown to be biocompatible and biodegradable and therefore appropriate for use *in vivo*. Furthermore, the storage and loss moduli of the hydrogels could be tuned (by varying the concentration of αCD) between a fraction of a kPa to a few 100 kPa, a range that coincides with the moduli of cells and human soft tissues. This property would allow for this hydrogel to be used *in vivo* with maximal mechanical compatibility with human soft tissues. *In vitro* experiments showed that the hydrogel demonstrated a linear mass erosion profile and a biphasic drug (doxorubicin) release profile: Phase I was primarily driven by diffusion and Phase II was driven by hydrogel erosion. The diffusion mechanism was modeled with the First Order equation and the erosion mechanism with the Hopfenberg equation. This established fitting model could be used to predict releases with other drugs and estimate the composition of the hydrogel required to achieve a desired release rate.

## 1. Introduction

Hydrogels of various properties is a topic that has been highly researched in the last few decades [1,2,3,4,5,6,7,8,9,10,11,12]. Due to their mechanical properties and high water content, they are predominantly used in biomedical applications [13,14,15,16,17,18,19]. Current applications in the market include dressings for wounds, such as the Kikgel, which is a cross-linking of synthetic and natural polymers [20], soft contact lenses out of silicone hydrogels [21] and even neural prosthetics using polypyrrole grown on alginate hydrogel scaffold [22]. PGS-PEGMEMA/α-cyclodextrin (αCD) hydrogels have properties that make them suitable candidates for the future development of such applications as well.

Injectable sustained-release drug delivery systems have been well-researched in the past decade, with some progress made, and a number of issues have been raised on the matter. Thermoplastic pastes like polylactic acid (PLA), poly(lactic-*co*-glycolic acid)(PLGA) and polycaprolactone (PCL) are capable of releasing Taxol™ for more than two months (greater than 60 days) [23]. One great disadvantage, however, is that they have to be heated up to a temperature of minimum 60 °C during injection. This hyperthermic treatment causes necrosis and tissue scarring, causing severe pain for the patient [23,24]. The blend of polymers in the polymeric paste also reduces the release rate of the gel, resulting in suboptimal treatment outcomes [25]. Other gel-like materials such as gelatin-agar [26], starch-carboxylmethyl cellulose or hyaluronic acid-methylcellulose [27] form a hydrogen bonded cross-linked hydrogel that is injectable [28]. They have excellent biocompatibility but disintegrate in a matter of hours due to the quick influx of water into the gel from the body [28]. This property thus limits these injectable gels to being suitable only in short, rapid drug release scenarios.

There is also a group of hydrogels that gel *in-situ*, after injecting the precursors of the hydrogel into the body. Alginate gels naturally occur upon contact with Ca^2+^, and researchers have hence designed thermoresponsive vesicles containing Ca^2+^ and co-delivered them with sodium alginate and the drug [29,30,31]. Upon heating to body temperature, these vesicles release Ca^2+^ and form a hydrogel matrix with alginate, enmeshing the drug for slow-release. However, as it takes a few hours for the alginate to gel, there is an initial burst of drug release which may bring about side effects from the drug [23,29,30,31]. Other methods such as solvent-removal *in-situ* polymer precipitation of poly-dl-lactide (PDLLA), PCL and PLA in physiologically compatible solvents like dimethyl sulfoxide (DMSO) or *N*-methyl-2-pyrrolidone (NMP) also face similar issues on the initial burst of drug delivery as it takes time for polymers to precipitate *in vivo* [23,32]. DMSO and NMP solvents are also toxic, in particular, myotoxic [23,33,34].

PEG has been commonly known to have the capacity of forming inclusion complexes with α-cyclodextrin via supramolecular forces [35,36,37]. As such, there have been previous attempts in synthesizing copolymers with grafted PEG segments to allow for the formation of supramolecular hydrogels. Ren *et al*. have reported in their studies the use of ATRP and free radical polymerization (FRP) in the synthesis of PPEGMA-*co*-PDMA copolymers that were able to form inclusion complexes with α-cyclodextrin in aqueous solution. This allowed them to achieve completely reversible temperature and pH dual-responsive supramolecular hydrogels [38].

In this case, we have managed to create a PGS-PEGMEMA/αCD supramolecular hydrogel that exhibited a low minimum gelation concentration of about 5%, rapid gelation (less than 3 minutes according to gel inversion experiment) and rapid self-healing ability with a modulus that is comparable to that of human soft tissue [5]. These properties make it a perfect candidate as an injectable drug delivery system. In this paper, we showed that the PGS-PEGMEMA/αCD hydrogel is biocompatible, biodegradable, and is capable of providing a sustained release to a chemotherapeutic drug without the initial burst effect.

## 2. Methods

### 2.1. Chemicals and Materials

Glycerol, sebacic acid, triethylamine (TEA), anhydrous tetrahydrofuran (THF), bromoisobutyryl bromide (BIBB), polyethylene glycol methyl ether methacrylate (PEGMEMA), 1,1,4,7,10,10-Hexamethyltriethylenetetramine (HMTETA), copper (I) bromide, doxorubicin, triethylamine (TEA), ethyl α-bromoisobutyrate (EBIB) and α-cyclodextrin (αCD) were purchased from Sigma-Aldrich, Nucleos, Singapore. Dulbecco’s modified eagle medium (DMEM), MTT and cell culture reagents was purchased from Life Technologies (Carlsbad, CA, USA).

### 2.2. Synthesis of PGS-PEGMEMA

An equimolar of sebacic acid and glycerol was weighed and mixed at 120 °C under N_2_ for 24 h. The mixture was reacted at 120 °C under 30 mTorr vacuum for 48 h to yield the PGS prepolymer. The PGS prepolymer was reacted with 5 molar ratios of BIBB and 1.2 molar ratio of TEA in THF overnight at room temperature to form PGS-Br, the ATRP macroinitiator. The PGS-Br macroinitiator (0.16 g) was then polymerized via ATRP with PEGMEMA (8 g) as the monomers to form PGS-PEGMEMA. Gel permeation chromatography (GPC) (Waters 2690, Milford, MA, USA.) with chloroform solvent was used to quantify the molecular weight of the synthesized polymer. GPC was calibrated with a set of polystyrene polymers of known molecular weights.

### 2.3. Preparation of the PGS-PEGMEMA/αCD Hydrogel

The hydrogels were prepared by mixing the different quantities of stock solution of 20% αCD and the stock solution of 10% PGS-PEGMEMA (*w*/*v* %) in PBS and letting the mixture set to form a white supramolecular hydrogel. All hydrogels were prepared with 2% polymer and a varying amount of αCD at 5%, 9%, and 13% (*w*/*v* %) in PBS, unless stated otherwise.

### 2.4. Biocompatibility of PGS-PEGMEMA

The cytotoxicity of the polymers was evaluated using the MTT assay in CCD-112CoN human fibroblast cell lines. PEG20kDa was used as a frame of reference to evaluate the relative biocompatibility of PGS-PEGMEMA. The cells were cultured in Dulbecco’s modified eagle medium (DMEM), supplemented with 10% fetal bovine serum (FBS), 100 units/mL of penicillin and 100 μg/mL of streptomycin at 37 °C under 5% CO_2_, and 95% relative humidity atmosphere. The cells were seeded in a 96-well plate at a density of 2 × 10^4^ cells/well and incubated for 24 h. The culture medium was then replaced with fresh culture medium containing polymer solutions of PEG20kDa and PGS-PEGMEMA at concentrations 31.3–1000 µg/mL. The control wells did not contain any polymer. MTT assay was used to evaluate the cell viability after the cells were incubated in the polymers of various concentrations over 24 h. The absorbance of the MTT crystals was measured using a microplate reader (Infinite M200, Tecan, Männedorf, Switzerland) at a wavelength of 570 nm. The cell viability at each polymer concentration was averaged within its duplicates and normalized to the averaged value of the control wells to obtain the cell viability percentages.

### 2.5. Biodegradability of PGS-PEGMEMA

Twenty milligrams of PGS-PEGMEMA was dissolved in 5 mL of PBS (pH 7.4), pH 2 buffer (50 mL 0.2 M potassium chloride + 13 mL 0.2 M hydrochloric acid) and pH 13 buffer (50 mL 0.2 M potassium chloride + 132 mL 0.2 M sodium hydroxide), and left to degrade at 37 °C. The polymer molecular weight was evaluated at various time points using a gel permeation chromatography (GPC) (Waters 2690).

### 2.6. Rheology of the PGS-PEGMEMA/αCD Hydrogel

PGS-PEGMEMA hydrogel samples were prepared as described above, and sheared on a rheometer (TA Discovery HR-3, New Castle, DL, USA) across different oscillation strain % and frequencies at 37 °C. Each hydrogel was loaded and conditioned at 37 °C for 300 s, followed by a logarithmic sweep of strain% from 0.01% to 10% at a constant frequency of 0.1 Hz at 37 °C. The sample then remained at 37 °C undisturbed for another 300 s before being subjected to a logarithmic sweep of frequencies from 0.01 to 100 Hz at a constant strain of 0.01% at 37 °C. The storage and loss moduli were plotted against strain % and frequency, respectively. In the self-healing test, the hydrogel was first conditioned at 37 °C for 300 s, followed by 10 cycles of alternations of 600 s at a low strain of 0.01% and 100 s at a high strain of 10%. The loss and storage moduli were plotted with time.

### 2.7. Scanning Electron Microscopy of the PGS-PEGMEMA/αCD Hydrogel

Low vacuum SEM (JEOL LV SEM 6360LA, Akishima, Tokyo, Japan) was used to image on the hydrogel samples with 2% polymer + 5%, 9% or 13% αCD. A thin layer of each gel was smeared onto the gold platform before imaging directly at 600× magnification with an accelerating voltage of 10 kV.

### 2.8. Hydrogel Erosion of the PGS-PEGMEMA/αCD Hydrogel

One milliliter of each of the 2% PEGMEMA + 5%, 9%, or 13% αCD (*w*/*v* %) hydrogels was prepared and left to erode in 1 mL of PBS at 37 °C. One hundred microliters of sample was collected and fresh PBS solution of the same volume was replaced once every 2 h for the first 6 h. Subsequent time points were taken twice daily, with 900 µL of erosion solution withdrawn (and replaced with equivolume of fresh PBS) and 500 µL withdrawn (and replaced with equivolume of fresh PBS) at every other time point. This procedure was repeated until the gels were fully eroded. Images of the eroding hydrogels were taken at several time points to show physical changes to the hydrogels. The samples collected at each time point and a 1 mL aliquot of fresh PBS were dried in an oven. The amount of hydrogel eroded during each time point was calculated by subtracting the weight of dried PBS of the corresponding volume from the weight of the dried samples of each time point. All experiments were conducted in duplicates and averaged.

### 2.9. Drug Release from the PGS-PEGMEMA/αCD Hydrogel

A stock solution of doxorubicin (5 mg/mL) was prepared and neutralized with triethylamine (TEA). 1 mL hydrogels of 2% PEGMEMA + 5%, 9%, or 13% αCD (*w*/*v* %) were prepared and were incorporated with payloads of either 400 or 500 µg of doxorubicin. The hydrogels were covered in aluminum foil to prevent photo bleaching of the doxorubicin, and released in 1 mL of PBS solution at 37 °C. The schedule for collecting the time points was the same as that in the erosion experiment (100 µL for the first 6 h and 900 and 500 µL for the subsequent time points). One hundred microliters at each time point was added to a 96-well plate and its absorbance was read on a plate reader (Infinite M200, Tecan, Männedorf, Switzerland) at 480 nm. A calibration curve of 10–500 µg/mL of doxorubicin was prepared with each round of drug release quantification. Intrapolation of the calibration curve was used to determine the amount of doxorubicin released at each time point. The fraction of mass release as compared to the payload was plotted over time. All experiments were conducted in duplicates and averaged.

## 3. Results

### 3.1. Synthesis and PGS-PEGMEMA/αCD Hydrogel Preparation

We synthesized two polymers with similar molecular weights and named them PGS-PEGMEMA 1 and 2 (Table 1). Due to the similar molecular weights of the two PGS-PEGMEMAs, we expected their properties to be similar, and will act as a proof of reproducibility and consistency for each other.

**Figure 1:**
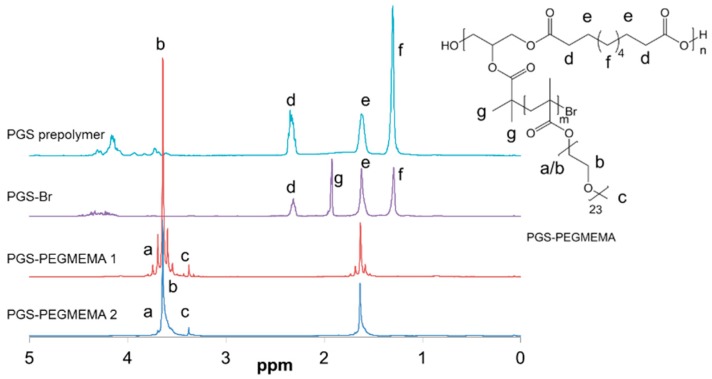
NMR spectrums showing the structures and purity of all intermediates and end products of the synthesis of PGS-PEGMEMA.

The synthesis of the PGS-PEGMEMA polymers was verified step-wise using NMR which showed that all intermediates of the reaction were successful and purified (Figure 1). In particular, the peak corresponding to the bromoisobutyrate group (peak g) verified the successful synthesis of the PGS-Br ATRP initiator. By taking the ratio of the area-under-curve between peak g and peak d, we calculated that the degree of brominization is approximately 94%. Due to the large proportion of PEGMEMA present in the PGS-PEGMEMA macromolecules, peaks corresponding to PGS were no longer visible in the NMR spectrums of PGS-PEGMEMA 1 and 2. However, strong peaks corresponding to the ether groups in the PEGMEMA verified the successful polymerization of PEGMEMA. The target molecular weight after ATRP is about 200,000 g/mol. However, due to the high density and therefore the proximity of reactive groups on the PGS backbone, it is likely that not all groups underwent ATRP, resulting in a lower-than-expected molecular weight.

**Figure 2:**
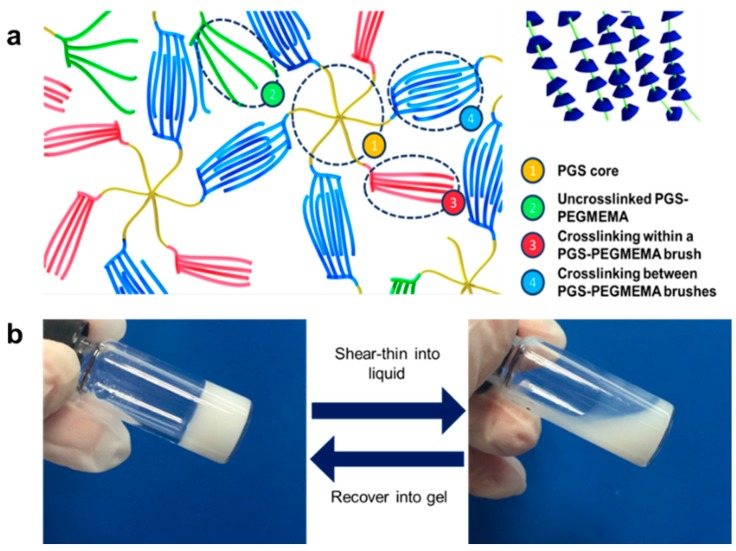
(**a**) Schematic showing the structure of the PGS-PEGMEMA/αCD hydrogel. Each arm of the multi-armed PGS core (1) was polymerized with a “brush-structured” PEGMEMA. PEGMEMA was capable of forming inclusion complexes with αCD. The inset showed the threading of αCD onto each brush of PGS-PEGMEMA. These brushes with columns of stacked αCD formed polypseudorotaxanes which could crosslink to other polypseudorotaxanes to form intra- (3) or inter- (4) polymer hydrogen bonding by proximity. Some brushes may remain uncrosslinked (2); (**b**) Picture showing the shear-thinning of the PGS-PEGMEMA/αCD hydrogel into a liquid. The gel set back into a gel if left to sit.

The PGS prepolymer had many unreacted hydroxyl groups (contributed by the glycerol monomers) per molecule which were exploited for the bromination and the subsequent grafting of PEGMEMA. The product of synthesis could be interpreted as a biodegradable core molecule (PGS) with multiple arms of PEGMEMA molecular “brushes” extending out of the core molecule (Figure 2a). During the preparation of the hydrogel, after the mixing of the dissolved PGS-PEGMEMA and αCD, the mixture started off as a clear solution. Upon setting for a short period of time, a white opaque PGS-PEGMEMA/αCD hydrogel was formed. αCD is known to form inclusion complexes with PEG by threading onto the PEG chains. In the PGS-PEGMEMA/αCD hydrogel, setting the mixture allowed time for αCD to be threaded onto PGS-PEGMEMA chains to form columns of stacked αCD, or polypseudorotaxanes (Figure 2a inset). These polypseudorotaxanes then crosslinked with each other, both within the same PGS-PEGMEMA brush (Figure 2a3) or between PGS-PEGMEMA brushes (Figure 2a4), via hydrogen bonding to form the hydrogel matrix. Some brushes may remain uncrosslinked (Figure 2a2).

As this hydrogel matrix was formed by supramolecular forces (hydrogen bonding between polypsuedorotaxanes and weak hydrophobic interactions in the inclusion complexes), these bonds could be broken and re-formed relatively easily. This contributed towards giving this hydrogel a thixotropic characteristic where the application of a force was able to shear thin the hydrogel into a liquid. Figure 2b showed that after agitation of the hydrogel with a spatula, the hydrogel shear-thinned into a flowable liquid. This liquid could recover into a non-flowable hydrogel state if left undisturbed for a period of time as the supramolecular bonds re-formed the hydrogel matrix. This shearing and recovery process was fully reversible, giving the hydrogel its ability to “self-heal”.

### 3.2. Biocompatibility of PGS-PEGMEMA

**Figure 3:**
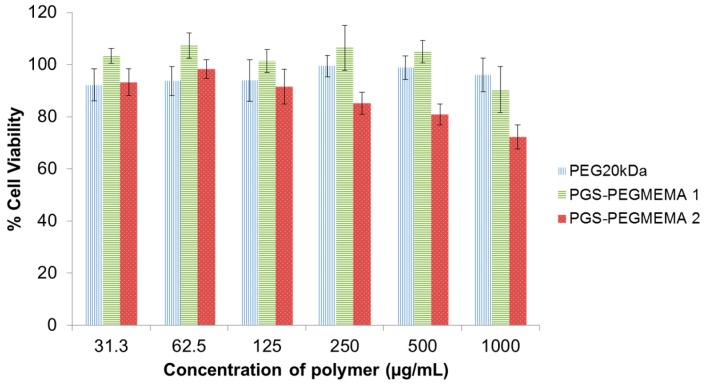
Biocompatibility of PGS-PEGMEMA 1 and 2 as compared to the control of PEG20kDa. Both PGS-PEGMEMA polymers showed very good biocompatibility with the human fibroblast cells.

The biocompatibility of PGS-PEGMEMA 1 and 2 in CCD-112CoN human fibroblast cells were compared with PEG20kDa. PEG was chosen as it is widely known to be highly biocompatible and would be a good benchmark for a biocompatible material. The results after incubation with human fibroblast cells over 24 h showed that PGS-PEGMEMA 1 was as biocompatible, if not more biocompatible, than the control of PEG20kDa at all tested polymer concentrations (31.3–1000 µg/mL) (Figure 3). PGS-PEGMEMA 2 exhibited slightly reduced cell viability with increasing polymer concentration from near 100% to approximately 75%. However, at concentrations below 500 µg/mL (equivalent of 50% *w*/*v*), PGS-PEGMEMA 2 was still able to offer a high biocompatibility of greater than 80%, which was comparable to the PEG20kDa control sample.

### 3.3. Biodegradability of PGS-PEGMEMA

Biodegradation experiment was conducted on PGS-PEGMEMA 2 polymer. It showed no significant change in the molecular weight of the polymer over a period of six weeks at neutral pH. This was expected as it had been reported that the ester bonds of PGS degrades significantly faster *in vivo* than *in vitro* [39,40]. To accelerate the degradation to demonstrate its biodegradability, PGS-PEGMEMA 2 was degraded under both acidic (pH2) and basic (pH13) environments at 37 °C. The weight-averaged molecular weight (*M*_w_) of the polymer plunged from 120 kDa to 57 kDa and 50 kDa in acidic and basic buffers, respectively, in merely two weeks (Table 2). This showed that PGS-PEGMEMA was capable of being hydrolyzed. However, a more biologically-relevant environment should be conducted to further evaluate its biodegradation properties.

### 3.4. Rheology of the PGS-PEGMEMA/αCD Hydrogel

**Figure 4:**
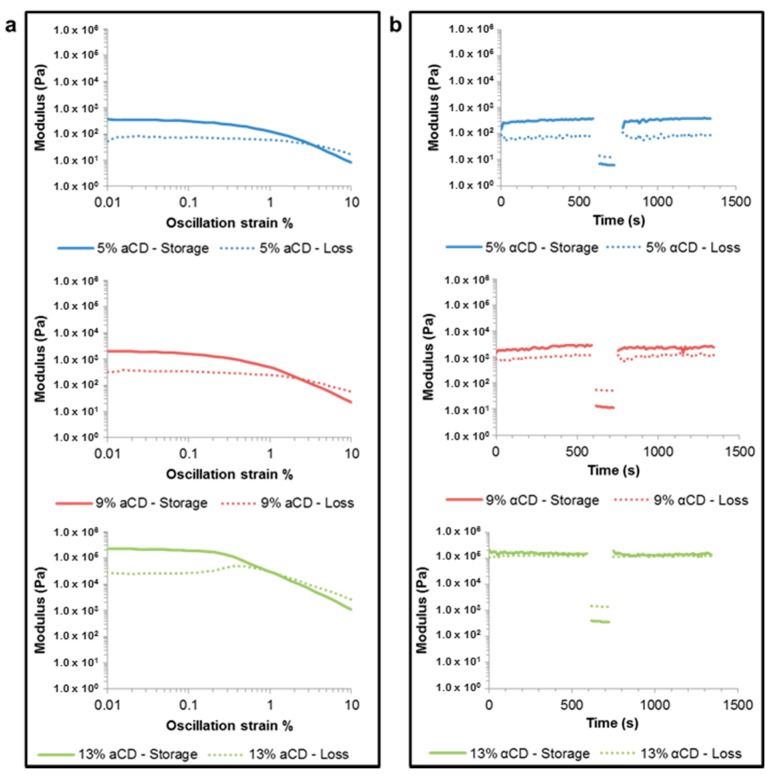
Rheology performed on hydrogels formed with 2% PGS-PEGMEMA 2 and varying concentrations of αCD: 5% (**top**), 9% (**middle**) and 13% (**bottom**). The moduli of the hydrogels increased as the concentration of αCD increased. (**a**) As the oscillation strain % increased from 0.01% to 10%, the hydrogel changed from a gel (*G*’ > *G*’’) to a liquid (*G*’ < *G*’’). This demonstrated that the hydrogel is thixotropic as the hydrogel could be sheared into a liquid upon the application of a high shear strain; (**b**) The strain % was alternated between 0.01% and 10% and back to 0.01%. The hydrogel changed from a gel state (*G*’ > *G*’’) to a liquid state (*G*’ < *G*’’) when sheared with 10% strain. The liquid then “self-healed” back into its gel state when the shear strain was reduced back to 0.01%.

Rheology test was performed to characterize the shear thinning and self-healing properties of the PGS-PEGMEMA/αCD hydrogels. The oscillation strain sweep was performed on the hydrogels prepared with 2% PGS-PEGMEMA 2 and 5%, 9% or 13% αCD to investigate the response of the hydrogels to shear strains in the range of 0.01%–10%. Figure 4a showed that at low shear strain, the storage modulus (*G*’) was greater than the loss modulus (*G*’’), which was consistent with the definition of a gel. As the shear strain % increased, *G*’ started to decrease at a rate faster than that of *G*’’. At a higher strain percentage of greater than 1%, the *G*’ dropped below that of *G*’’ and the gel turned into a liquid. This response of the hydrogel to shear strain demonstrated its thixotropic property. The modulus of the hydrogel increased with increasing αCD concentration, due to the increased number of hydrogen bonds between the polypseudorotaxanes.

The oscillation frequency sweep of the same hydrogels from 0.01 to 10 Hz at a shear strain of 0.01% showed that the oscillation frequency did not affect the integrity of the hydrogel significantly (Supplementary Figure S1).

In the self-healing test (Figure 4b), the rheology results showed that the PGS-PEGMEMA 2/αCD hydrogel was capable of recovering, or “self-healing” back into a gel state after being shear-thinned at 10% strain. *G*’ was higher than *G*” initially during the 600 s of 0.01% strain and the gel was sheared into a liquid where *G*’ < *G*” during the 100 s of 10% strain. The *G*’ and *G*” were then reversed back into *G*’ > *G*” quickly when the strain was reduced back to 0.01%. This switching and reversing of the relationship between *G*’ and *G*” was repeatable for a minimum of 10 shear-recovery cycles, proving its self-healing property. This self-healing property, along with its superior biocompatibility and biodegradability, made it a good candidate for an injectable drug delivery matrix.

### 3.5. Scanning Electron Microscopy of the PGS-PEGMEMA/αCD Hydrogel

The SEM images elucidated the microstructures of the PGS-PEGMEMA/αCD supramolecular hydrogels with different αCD concentrations (Table 3). As the αCD concentrations increased, the matrix of the hydrogel became denser and the pore size was reduced. This trend was shown to be consistent between the hydrogels formed with both PGS-PEGMEMA 1 and 2.

### 3.6. Hydrogel Erosion of the PGS-PEGMEMA/αCD Hydrogel

**Figure 5:**
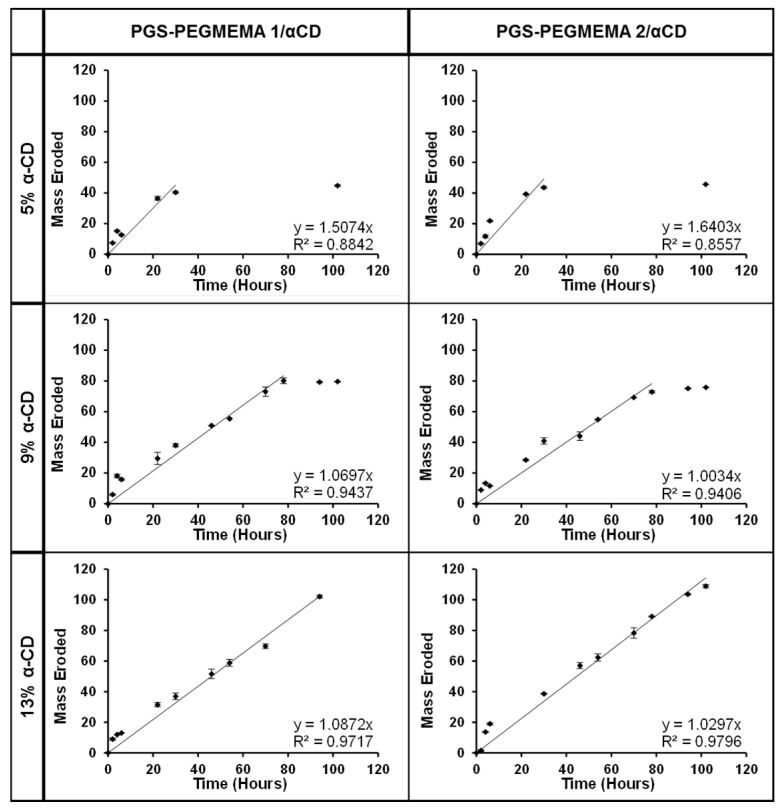
The erosion profile of hydrogels prepared with PGS-PEGMEMA 1 and 2 with αCD concentrations of 5%, 9% and 13%. The erosion rate was rather consistent independent of the αCD concentrations.

The mass erosion profile of hydrogel was plotted against time for the hydrogel formed with each of PGS-PEGMEMA 1 and PGS-PEGMEMA 2, with various αCD concentrations. There was no significant difference between the two hydrogels. The rates of erosion were fairly consistent (independent of the αCD concentrations) at an average erosion rate of 1.22 ± 0.25 mg/h and 1.22 ± 0.36 mg/h for PGS-PEGMEMA 1 and 2, respectively. The erosion was relatively linear over time with R^2^ values close to 1 (Figure 5). The rate of the percentage of hydrogel eroded over time decreased with increasing αCD concentrations (Supplementary Figure S2).

**Figure 6:**
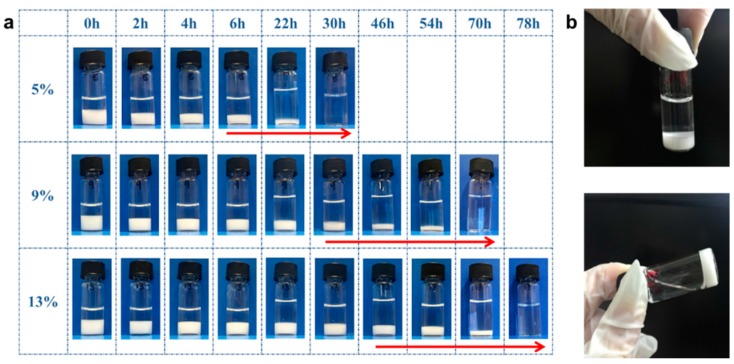
Images of the PGS-PEGMEMA1/αCD hydrogel. (**a**) Images taken at various time points for 5%, 9% and 13% αCD. The hydrogels went through two phases of erosion at all αCD concentrations: Phase I where there was no visible change to the gel and Phase II where there was a gradual one-dimensional erosion from the top surface. Phase II had been marked by the arrow; **(b**) Image taken at 54 h for αCD concentration of 13%. The hydrogel had started to erode but the un-eroded portion was able to maintain its integrity and remain as a gel.

Images of the eroding hydrogels taken at various time points (Figure 6a) elucidated that the hydrogel eroded in a bi-phasic manner. Phase I involved a loss of mass (as elucidated in Figure 5) without any visible change to the hydrogels. In Phase II, the hydrogels underwent one-dimensional physical erosion and loss of volume from the top surface of the gel where it was in contact with the PBS solution. The arrows in Figure 6a demarcated the start and end of Phase II. The remaining uneroded part of the hydrogel maintained its integrity as demonstrated in Figure 6b which was taken at the 54 h time point. This implied that this hydrogel matrix could be considered a surface eroding matrix.

### 3.7. Drug Release from the PGS-PEGMEMA/αCD Hydrogel

**Figure 7:**
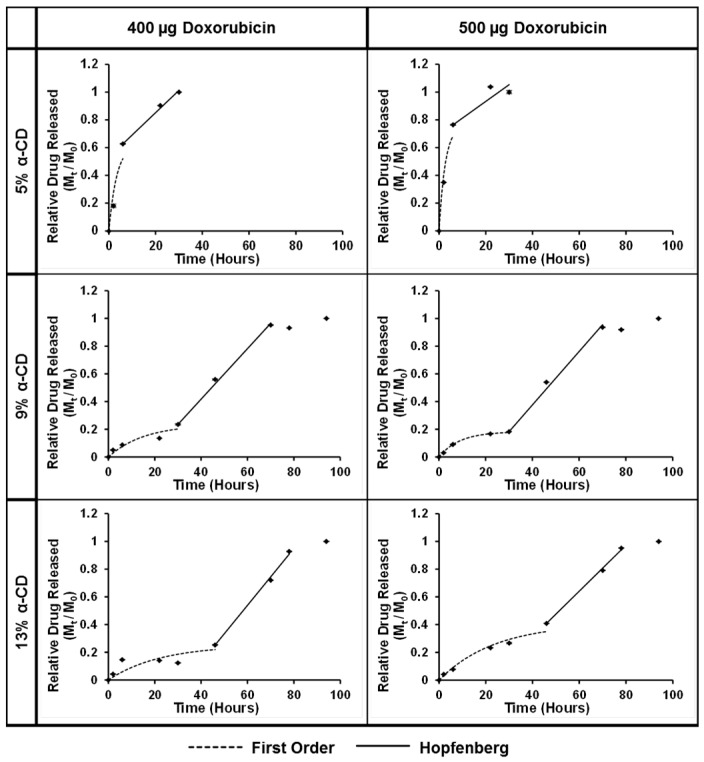
Drug release profile with model fitting for the PGS-PEGMEMA 2/αCD hydrogels with 2% polymer and 5%, 9% and 13% αCD with payloads of 400 and 500 µg. Phase I was fitted to the First Order Kinetic model (dotted) and Phase II was fitted to the Hopfenberg model (solid).

Drug release experiments were performed with PGS-PEGMEMA 2/αCD hydrogel (at 2% PGS-PEGMEMA 2 and 5%, 9% and 13% αCD) and doxorubicin with a payload of 400 µg and 500 µg in a 1 mL hydrogel. The release profiles of relative mass released ($\frac{M(t)}{M_{0}}$) over time in hours was plotted (Figure 7). Two phases of drug release were observed for the hydrogels, which coincided with the two phases observed in the erosion experiments. Phase II started at 6 h for 5% αCD, 30 h for 9% αCD and 46 h for 13% αCD, which corresponded exactly with the erosion profile.

In Phase I, since there was no significant physical change to the hydrogel matrix, we hypothesized that the release of doxorubicin occurred entirely via diffusion, driven by the concentration gradient of the drug between inside and outside of the hydrogel. This was justified when a good fit was obtained with the First Order Kinetics model as shown by the dotted curved lines (Figure 7).

**Figure 8:**
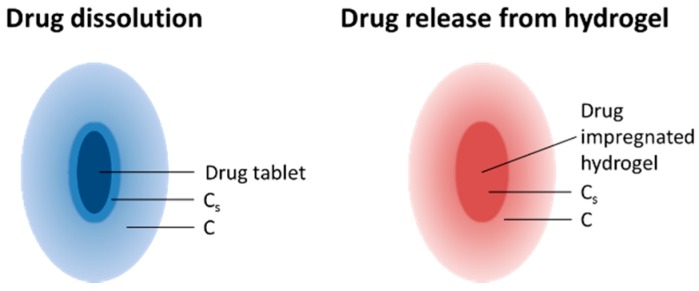
Schematic illustrating the differences in the terms C_s_ and C between the drug dissolution and drug release from a hydrogel used in the First Order Kinetic model.

The First Order Kinetics model, which was derived from the Noyes-Whitney equation, traditionally describes the dissolution of a drug [41,42].

(1) $$\frac{dC}{dt}=k(C_{s}-C)$$

where C is the concentration of solute in time t, $C_{s}$ is the solubility in the equilibrium and k is the first order proportionality constant. The driving force for the release is, therefore, $C_{s}-C$ (Figure 8). However, in this case of drug release from a hydrogel matrix with a constant sink condition (maintained by exchanging for fresh PBS at every time point), $C_{s}$ is the concentration of the drug inside the hydrogel and C≈0. The release rate, $\frac{dC}{dt}$, is therefore directly proportional to the concentration of the drug remaining inside the hydrogel. We could re-write the equation in the form:

(2) $$\frac{dC_{s}}{dt}=k_{1}C_{s}$$

where $C_{s}$ is the concentration of drug inside the hydrogel matrix and $k_{1}$ is the first order release constant for the hydrogel. This equation could be manipulated into

(3) $$C_{s}(t)=C_{0}e^{-k_{1}t}$$

where $C_{0}$ is the initial drug concentration in the hydrogel matrix at t = 0. The mass of drug released over time, $M_{t}(t)$, would therefore be:

$M_{t}(t)=Payload-C_{s}(t)\times$ volume of hydrogel

(4) $$M_{t}(t)=M_{0}(1-e^{-k_{1}t})$$

(5) $$\frac{M_{t}}{M_{0}}=1-e^{-k_{1}t}$$

where $M_{0}$ is the payload of the hydrogel. This equation was used to fit to the drug release profile in Phase I. Note that the volume of hydrogel was constant because there was no visible change to the hydrogel volume during Phase I.

In Phase II, physical erosion of the hydrogel matrix started to take place as the matrix structure was gradually getting disrupted. This resulted in a different mechanism of release via matrix erosion. The data in Phase II was fitted to the Hopfenberg model, as delineated by the solid line in Figure 7. All the fitting parameters were tabulated in Table 4.

The Hopfenberg model describes drug release from a surface eroding polymer where the release surface area is constant throughout the release [43,44]:

(6) $$\frac{M_{t}(t)}{M_{\infty }}=1-(1-\frac{k_{0}}{C_{L}a}t)$$

where $M_{t}$ is the mass of drug released at time *t*, $M_{\infty }$ is the mass of drug released at time infinity (which would be theoretically equivalent to the payload, $M_{0}$), $k_{0}$ is the zero order rate constant, $C_{L}$ is the initial drug concentration in the matrix and a is the initial radius for a sphere or cylinder or half-thickness for a slab.

The erosion experiment concluded that the PGS-PEGMEMA/αCD hydrogel was eroding from only the top surface with a constant area of release. The Hopfenberg was therefore an appropriate model for modeling an erosion-driven drug release. As the erosion of the hydrogel took place on a flat surface instead of a cylindrical or spherical surface, we defined *n* = 1 as that of a slab. The Hopfenberg equation could therefore be simplified to:

(7) $$\frac{M_{t}}{M_{0}}=k_{2}t$$

where $k_{2}$ is the Hopfenberg constant for Phase II. This equation was used to fit to the drug release profile in Phase II.

## 4. Discussion

We demonstrated that our PGS-PEGMEMA/αCD hydrogel was biocompatible with cell viability with human fibroblast cells of at least 80%. This was comparable with that of PEG20kDa, which served as the biocompatible bench mark. The hydrogel was also biodegradable under accelerated degradation conditions of acidic and basic environments. Further degradation experiments in a more *in vivo*-like environment would allow for a better evaluation of its degradation properties.

The rheology data showed that the mechanical strength of the PGS-PEGMEMA/αCD increased with increasing αCD concentration. As the amount of αCD increased, the number of polypseudorotaxanes on the PGS-PEGMEMA brushes increased, resulting in more hydrogen bonds between the polypseudorotaxanes. This gave rise to a larger number of, and therefore stronger, crosslinks in the hydrogel and hence the higher moduli. A modulus of as high as 100 kPa could be achieved with only 2% (*w*/*v*) of PGS-PEGMEMA and 13% (*w*/*v*) of αCD.

We also demonstrated that the moduli of the PGS-PEGMEMA/αCD hydrogels could easily be tuned from a fraction of kPa to ~100 kPa by adjusting the αCD concentration. This range of moduli is very physiologically relevant as biological cells also have moduli that range from ~0.1 kPa (in endothelial cells) to a few hundred kPa (in cardiovascular cells) [45,46]. Our hydrogel can therefore be easily tailored to match the moduli required by specific intended applications. The range of moduli has the potential to be extended by further investigating the effect of PGS-PEGMEMA concentration on the moduli of the hydrogel.

The hydrogel was also capable of shear thinning upon the application of a shear force. Our previous literature had reported that this hydrogel was capable of “self-healing”—the ability to revert back into a hydrogel quickly after it had been shear-thinned into a liquid. This interesting thixotropic property made this hydrogel a good candidate for use as an injectable matrix for drug delivery. The idea was to preload the drug-impregnated hydrogel into a syringe such that the pressure from the syringe plunger would shear the hydrogel into a liquid to be passed through the needle into human tissue. Upon injection, the liquid would quickly recover back into a hydrogel that would be capable of localized sustained release of the drug into the area of injection. This potential application was then evaluated with the erosion experiment and the drug delivery study with doxorubicin as a model drug.

The mass erosion profile of the hydrogel exhibited a linear mass loss profile which could be divided into two phases—Phase I where there was no visible change to the hydrogel, and Phase II where the hydrogel started to erode in one-dimension. During Phase I, the main loss of mass was probably due to the diffusion of the free αCD molecules (that did not form inclusion complexes with PGS-PEGMEMA brushes) into the PBS surrounding the hydrogel. As this process did not affect the crosslinking of the hydrogel matrix significantly, there was no visible change to the hydrogel. Phase II, however, was when the threaded αCD started to undergo unthreading and dissolution into the surrounding PBS, disrupting the hydrogel matrix. This led to a visible reduction of the volume over time as the hydrogel matrix was gradually lost. The rate of erosion of the hydrogel was therefore limited by the rate of unthreading and dissolution of αCD, independent of the hydrogel matrix density and strength, at 1.22 ± 0.25 and 1.22 ± 0.36 mg/h for PGS-PEGMEMA 1 and 2, respectively. As the erosion happened only on the top surface where the hydrogel was in contact with PBS and the rest of the hydrogel remained intact, the PGS-PEGMEMA/αCD hydrogel could be considered to be a surface eroding matrix.

The drug release profile demonstrated a biphasic characteristic that coincided with the erosion profile. It was observed that the first order release constant, *k*_1_, decreased with increasing αCD concentration. As the αCD concentration increased, the matrix density increased as shown by SEM images. The rate of drug diffusion therefore slowed down, resulting in a drop in *k*_1_ value. The *k*_1_ values in hydrogels with a payload of 500 µg of doxorubicin were also generally higher than that with a payload of 400 µg at the same αCD concentration. This was coherent with the fact that a higher payload provided a steeper drug concentration gradient between the inside and outside of the hydrogel, which in turn acted as a stronger driving force for diffusion to take place, resulting in higher *k*_1_ values.

The Hopfenberg release constant in Phase II, *k*_2_, was relatively constant regardless of the αCD concentration or the payload. This was expected as the rate of release by matrix erosion would be proportional to the rate of erosion which was shown to be relatively constant for all concentrations of αCD.

This PGS-PEGMEMA/αCD hydrogel was able to drastically reduce the initial burst effect of many drug delivery matrices upon the increase of its αCD concentration. Compared to a hydrogel at 5% αCD, higher αCD concentrations allowed for a more sustained release of doxorubicin over a period of 60–80 h. Further studies on how the polymer concentration could improve the desired release profile could be conducted to optimize the PGS-PEGMEMA/αCD hydrogel for use as an injectable drug delivery matrix.

## Figures and Tables

**Table 1:** The molecular weights of PGS-PEGMEMA 1 and 2.

Sample	*M*_w_ (g/mol)	PDI
PGS prepolymer	17,700	4.34
PGS-PEGMEMA 1	118,000	3.17
PGS-PEGMEMA 2	123,000	3.46

**Table 2:** The weight averaged molecular weight (*M*_w_) of PGS-PEGMEMA 2 over a period of six weeks at 37 °C in acidic, basic and neutral pH. The *M*_w_ stayed relatively constant in neutral media (PBS) over the course of six weeks, but was degraded from 120 to 57 kDa and 50 kDa in acid and basic buffer solutions respectively in just two weeks.

Degradation duration	Neutral degradation		Acid degradation		Base degradation	
Weeks	*M*_w_ (g/mol)	PDI	*M*_w_ (g/mol)	PDI	*M*_w_ (g/mol)	PDI
0	123,000	3.46	123,000	3.46	123,000	3.46
2	92,000	2.68	57,000	2.03	50,000	1.86
3	123,000	2.68	54,000	2.19	47,000	1.99
6	148,000	2.49	57,000	1.99	47,000	2.11

**Table 3:** Scanning electron microscope (SEM) images of the hydrogel formed with 2% PGS-PEGMEMA and 5%, 9% and 13% of α-cyclodextrin. The hydrogel matrix density increased with increasing αCD concentrations for both PGS-PEGMEMA 1 and 2/αCD.

Polymers	5% α-cyclodextrin	9% α-cyclodextrin	13% α-cyclodextrin
PGS-PEGMEMA 1	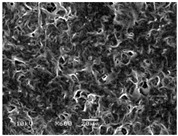	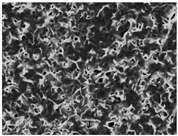	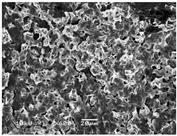
PGS-PEGMEMA 2	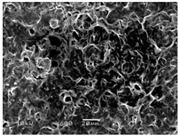	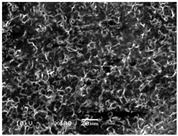	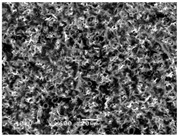

**Table 4:** The fit parameters, *k*_1_ and *k*_2_, representing the release constants of Phase I and Phase II respectively for PGS-PEGMEMA 2 at payloads of 400 and 500 µg. *k*_1_ decreased with increasing αCD concentrations, and the values of *k*_1_ were also higher for a payload of 500 µg compared to that of 400 µg at any particular αCD concentration. *k*_2_ was relatively constant regardless of payload and αCD concentrations.

**Phase I**	**PGS-PEGMEMA 2 (400 µg)**	***k*_1_**	***R*^2^**	**Phase II**	**PGS-PEGMEMA 2 (400 µg)**	***k*_2_**	***R*^2^**
	5%	0.295	0.899		5%	0.016	0.993
	9%	0.065	0.889		9%	0.018	0.995
	13%	0.045	0.659		13%	0.020	0.996
	PGS-PEGMEMA 2 (500 µg)				PGS-PEGMEMA 2 (500 µg)		
	5%	0.375	0.968		5%	0.012	0.785
	9%	0.113	0.997		9%	0.019	0.990
	13%	0.042	0.963		13%	0.017	0.997

## Supplementary Materials

The supplementary materials can be found at www.mdpi.com/2073-4360/8/4/130/s1.
