# Supplementary material for: A Thixotropic Polyglycerol Sebacate-Based Supramolecular Hydrogel as an Injectable Drug Delivery Matrix
Source: Polymers (Basel). 2016 Apr 7;8(4):130. doi: 10.3390/polym8040130 (PMC6432133; doi:10.3390/polym8040130)
Supplement: Supplementary file 1 [file polymers-08-00130-s001.pdf]

Supplementary Materials: A Thixotropic Polyglycerol Sebacate-Based Supramolecular Hydrogel as an Injectable Drug Delivery Matrix

Hongye Ye, Cally Owh, Shan Jiang, Cavin Zhen Quan Ng, Daniel Wirawan and Xian Jun Loh

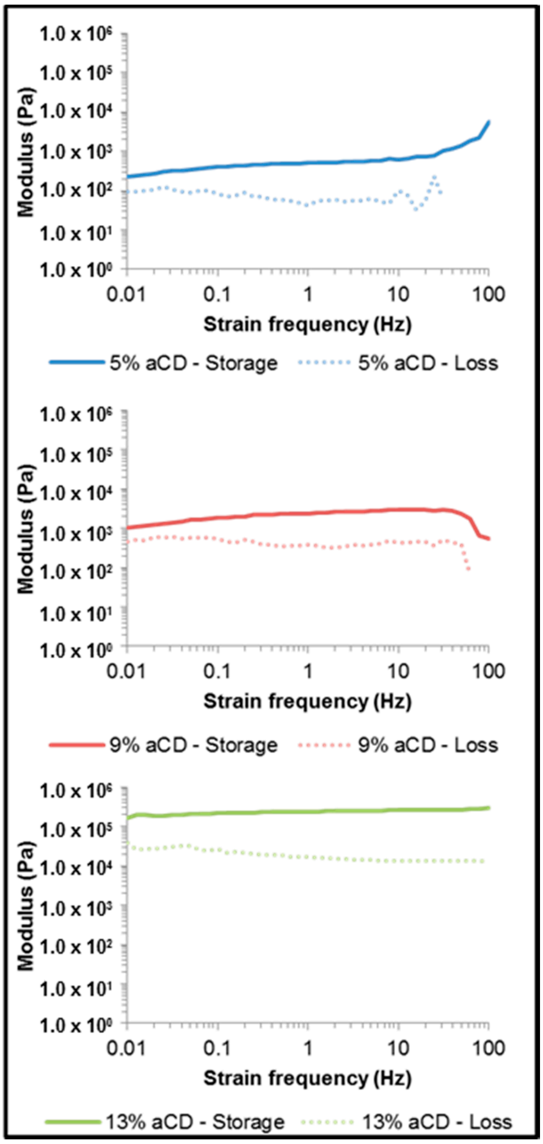

Figure S1. The oscillation frequency sweep in the range of 0.01–10 Hz at a shear strain of 0.01%. The hydrogels remained in a gel state ($G' > G''$) throughout the oscillation frequency sweep range and did not lose their integrity for all αCD concentrations.

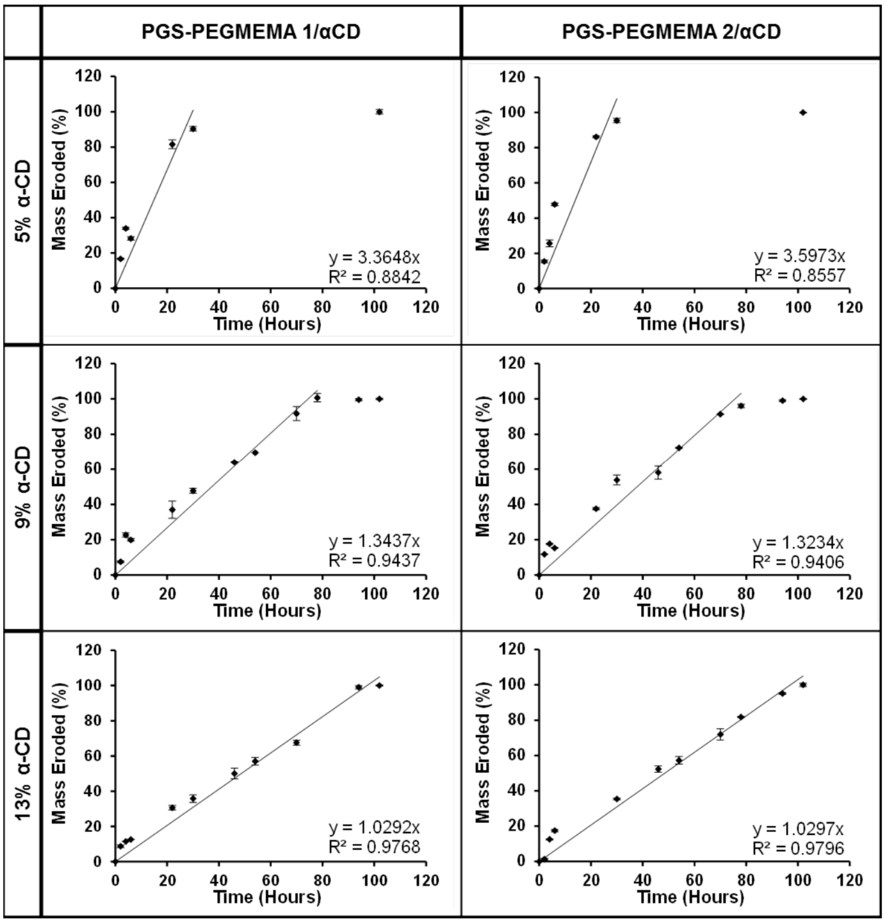

Figure S2. The percentage of mass of hydrogel eroded in PBS over time. The rate of the % mass eroded over time decreased with increasing αCD concentrations.

© 2016 by the authors; licensee MDPI, Basel, Switzerland. This article is an open access article distributed under the terms and conditions of the Creative Commons by Attribution (CC-BY) license (http://creativecommons.org/licenses/by/4.0/).
